# Targeting Nitrogen Metabolism and Transport Processes to Improve Plant Nitrogen Use Efficiency

**DOI:** 10.3389/fpls.2020.628366

**Published:** 2021-03-01

**Authors:** Samantha Vivia The, Rachel Snyder, Mechthild Tegeder

**Affiliations:** School of Biological Sciences, Washington State University, Pullman, WA, United States

**Keywords:** amino acid partitioning, nitrogen assimilation, crop improvement, nitrogen uptake and transport, nitrogen use efficiency, seed yield and quality, source and sink physiology, sustainable agriculture

## Abstract

In agricultural cropping systems, relatively large amounts of nitrogen (N) are applied for plant growth and development, and to achieve high yields. However, with increasing N application, plant N use efficiency generally decreases, which results in losses of N into the environment and subsequently detrimental consequences for both ecosystems and human health. A strategy for reducing N input and environmental losses while maintaining or increasing plant performance is the development of crops that effectively obtain, distribute, and utilize the available N. Generally, N is acquired from the soil in the inorganic forms of nitrate or ammonium and assimilated in roots or leaves as amino acids. The amino acids may be used within the source organs, but they are also the principal N compounds transported from source to sink in support of metabolism and growth. N uptake, synthesis of amino acids, and their partitioning within sources and toward sinks, as well as N utilization within sinks represent potential bottlenecks in the effective use of N for vegetative and reproductive growth. This review addresses recent discoveries in N metabolism and transport and their relevance for improving N use efficiency under high and low N conditions.

## Introduction

Nitrogen (N) is essential for general plant functions as it is a component of amino acids, which are the elemental units of protein and enzymes. Amino acids are also precursors or N donors for many fundamental compounds including nucleic acids, hormones, chlorophyll, ureides, and other metabolites required for primary metabolism and specialized biological functions, respectively (Lam et al., 1996; Lea and Ireland, 1999; Epstein and Bloom, 2005; Zrenner et al., 2006; Züst and Agrawal, 2016). In addition, amino acids are the main long-distance transport forms of N in plants (Urquhart and Joy, 1982; Turgeon and Wolf, 2009; Patrick, 2013; Tegeder and Hammes, 2018). While amino acids, peptides, or even proteins may be taken up from the soil (Chapin et al., 1993; Näsholm et al., 2009; Tegeder and Rentsch, 2010), nitrate and ammonium are often preferred (Crawford and Glass, 1998; Loqué and von Wirén, 2004; Krapp et al., 2014). However, the inorganic N is only usable by plants when it is assimilated into amino acids, a process that primarily occurs in source organs such as the roots or leaves (Andrews et al., 1992; Lam et al., 1996; Miller et al., 2007; Xu et al., 2012; Krapp, 2015) but can also take place in sinks like seeds (Rochat and Boutin, 1992; Weber et al., 1998; Chen et al., 2020).

Plants that preferentially reduce N in roots move the newly produced amino acids through the xylem to source leaves (Miflin and Lea, 1977; Schobert and Komor, 1990). In addition, some xylem amino acids may be transferred to the phloem along the translocation pathway for immediate N supply to fast-growing sinks (Pate et al., 1975; van Bel, 1984; Zhang et al., 2010). However, in many plant species nitrate is allocated predominantly to source leaves as photosynthesis provides the carbon backbone and the energy for amino acid synthesis (Lam et al., 1996; Nunes-Nesi et al., 2010; Tegeder and Masclaux-Daubresse, 2018). In leaves, xylem-derived or leaf-synthesized amino acids may be used for metabolism, especially for photosynthesis (Wallsgrove et al., 1983; Schulze-Siebert et al., 1984), transiently stored as amino acids or proteins to be used for reproductive development (Millard, 1988; Staswick, 1994; Liu et al., 2005; Nunes-Nesi et al., 2010), or they are actively loaded into the phloem and transported to growing sinks (Urquhart and Joy, 1982; Atkins and Beevers, 1990; Tegeder and Masclaux-Daubresse, 2018). Once arrived in sink organs, the phloem unloading and movement of amino acids to sink cells takes place (Knoblauch et al., 2016; Milne et al., 2018; Tegeder and Hammes, 2018). Within the seed coat of seed sinks, amino acid catabolism, transamination, and re-assimilation events may occur (Atkins et al., 1975; Rainbird et al., 1984; Weber et al., 1995; Gallardo et al., 2007) followed by an efflux of amino acids into the seed apoplastic space and their subsequent uptake by the embryo for development, metabolism, and synthesis of seed storage compounds (Patrick, 1997; Offler et al., 2003; Ladwig et al., 2012; Tegeder et al., 2013).

Over the past decades, large amounts of N fertilizer have been applied to maximize crop yields while causing negative consequences for ecosystems and human health due to N leaching from the soil or degradation and subsequently releasing into the atmosphere (Matson et al., 1997; Fowler et al., 2013; Kopittke et al., 2019). In addition, crop plants are often not efficient in acquiring and using the supplied N and may take up less than 50% of the N fertilizer (Raun and Johnson, 1999; Zhu et al., 2016). Consequently, increasing N fertilization does not necessarily result in a proportional increase in yield (Ju et al., 2004; Mueller et al., 2014; Zhu et al., 2016). Therefore, improvements in yield and N use efficiency (NUE) in conjunction with a reduction in N application and N losses into the environment are pressing goals for a sustainable agriculture (Zhang et al., 2015a; Anas et al., 2020). However, progress in NUE is an ambitious target as crop yields are influenced by numerous factors, including genetic parameters and their variation among and within species (G), environmental effects such as location, soil conditions, and climate (E), and agronomic technologies and management practices (M; e.g., type, timing, amount, and place of N application or weed and pest control) (Hatfield and Walthall, 2015; Nguyen et al., 2017; Martinez-Feria et al., 2018; Nguyen and Kant, 2018; Gramma et al., 2020; Jensen et al., 2020; Lemaire and Ciampitti, 2020; Plett et al., 2020). Only the combination or interaction of these factors (G × E × M) will allow for sustainable and secure crop production (Swarbreck et al., 2019; Cooper et al., 2020; Hawkesford and Riche, 2020; Peng et al., 2020).

At crop level, N use efficiency is defined as seed yield relative to N availability (Moll et al., 1982). It is comprised of N uptake efficiency (NUpE) or the proportion of N in the shoot relative to the N supply, and N utilization efficiency (NUtE), which describes the amount of shoot N used for seed production. Many genetic factors contribute to plant biomass production, yield, and NUE including those responsible for plant structure and architecture (Gifford et al., 2008; Xu et al., 2012; Li et al., 2015; Xie et al., 2017; Luo et al., 2020), as well as N root uptake, (re)assimilation, remobilization, partitioning, utilization, and signaling processes (Coque and Gallais, 2006; Chardon et al., 2010; McAllister et al., 2012; Han et al., 2015; Zhao et al., 2018; Griffiths and York, 2020). Numerous genes related to these developmental and physiological processes have been identified and their importance for plant growth, function, and seed yield has been demonstrated. We would like to refer the readers to some recent reviews that provide comprehensive overviews on the discoveries (Miller et al., 2007, 2008; Xu et al., 2012; Han et al., 2015; Mandal et al., 2018; Tegeder and Masclaux-Daubresse, 2018; Wang et al., 2018a; Raghuram and Sharma, 2019; Chen et al., 2020; Fernie et al., 2020; Fichtner et al., 2020; Li et al., 2020; Vidal et al., 2020). The current work will focus on new findings in N metabolism and transport, along with their associated genes and proteins, and analyze their role in improving NUE under high and low N conditions.

## Role of Inorganic Nitrogen Uptake and Partitioning in NUE

**Figure 1:**
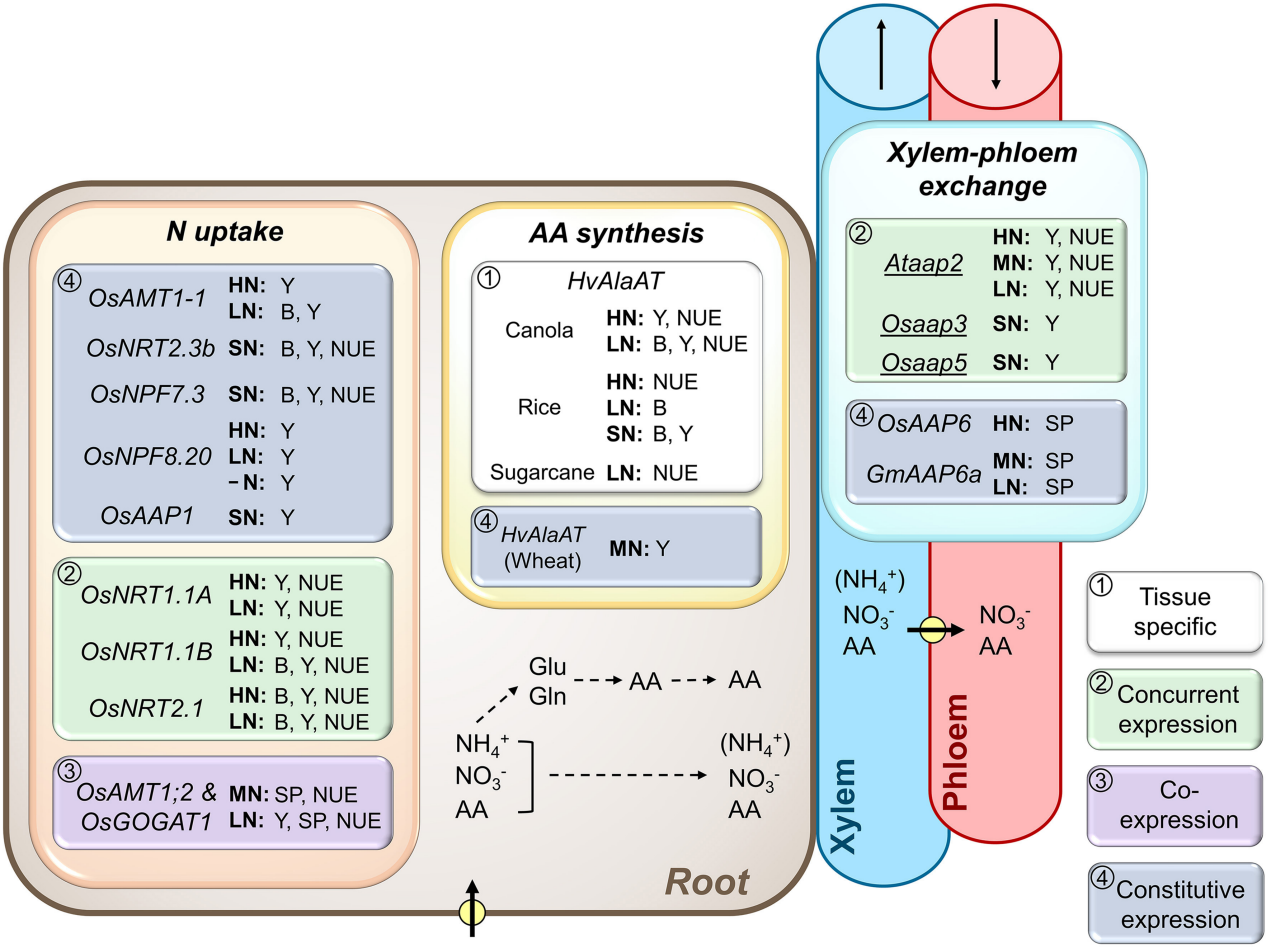
Importance of root N transport and metabolic processes, and xylem-phloem exchange along the transport pathway for plant biomass and yield production, and N use efficiency. Shown are ammonium (${\text{NH}}_{4}^{+}$), nitrate (${\text{NO}}_{3}^{-}$), and amino acid (AA) transporters, and AA synthesis genes that were successfully overexpressed (*UPPERCASE*) or repressed/knocked out (*lowercase and underlined*) and resulted in increased biomass (B), seed yield (Y), seed protein (SP), and/or plant N use efficiency (NUE) at varying N supply. As indicated by framed white, green, purple, or blue backgrounds, the transgenic strategies used either (1) tissue-specific gene manipulation, (2) concurrent expression of a particular gene in both root and shoot tissues (*OsNRT1.1A*, Wang et al., 2018b; *OsNRT1.1B*, Hu et al., 2015; *OsNRT2.1*, Chen et al., 2016), (3) co-expression of two genes in root and shoot tissues (*OsAMT1;2* and *OsGOGAT1*, Lee et al., 2020a), or (4) constitutive gene expression throughout the plant. Positive results were achieved with (a) native expression of ammonium transporters (*OsAMT1-1*, Ranathunge et al., 2014; *OsAMT1;2*, Lee et al., 2020a), nitrate transporters (*OsNRT1.1A*, Wang et al., 2018b; *OsNRT1.1B*, Hu et al., 2015; *OsNRT2.1*, Chen et al., 2016; *OsNRT2.3b*, Fan et al., 2016b; *OsNPF7.3*, Fang et al., 2017; *OsNPF8.20*, Fang et al., 2013), and amino acid transporters (*OsAAP1*, Ji et al., 2020; *OsAAP6*, Peng et al., 2014; *GmAAP6a*, Liu et al., 2020) in rice (*Os*), Arabidopsis (*At*), and soybean (*Gm*), (b) expression of a barley alanine aminotransferase (*HvAlaAT*) in canola (Good et al., 2007), rice (Shrawat et al., 2008; Beatty et al., 2013), sugarcane (Snyman et al., 2015), and wheat (Peña et al., 2017), and (c) by knocking out native/endogenous genes (*Ataap2*, Perchlik and Tegeder, 2018; *Osaap3*, Lu et al., 2018; *Osaap5*, Wang et al., 2019). Plants were grown under high (HN), moderate (MN), sufficient (SN; N supply was not specified) and low N (LN), or without N (-N). Pathways of N uptake and xylem-phloem transfer (arrows with circle), as well as N assimilation into glutamate (Glu) and glutamine (Gln), and the subsequent synthesis of other AA and their loading into the xylem are indicated. ${\text{NH}}_{4}^{+}$ may be transported in the xylem to the shoot, but in relatively low amounts (indicated by brackets). Arrows in xylem and phloem indicate the direction of translocation. For additional information see manuscript text.

**Figure 2:**
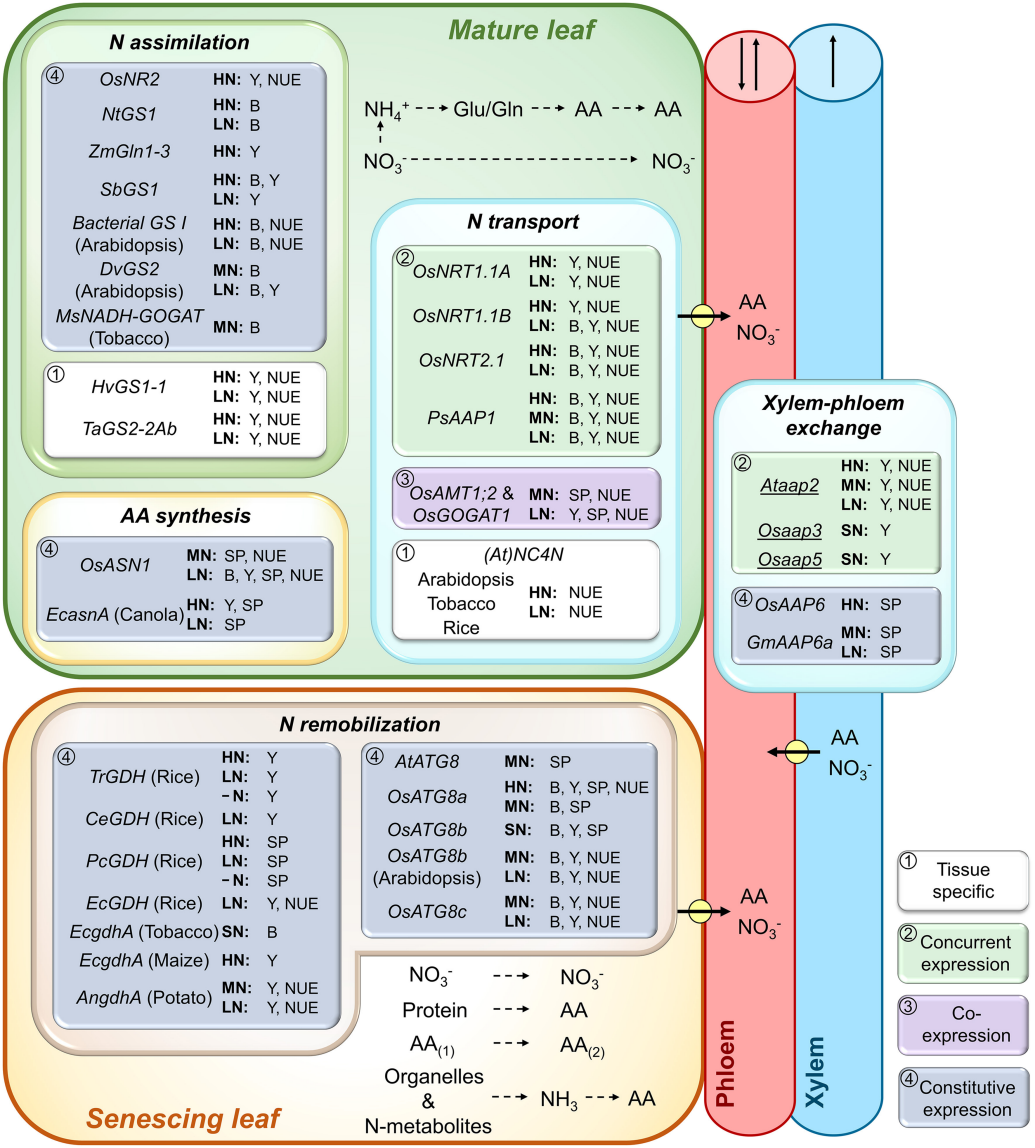
Role of leaf N (re)assimilation and remobilization, amino acid (AA) synthesis and N transport processes in plant biomass, yield production, and N use efficiency. Genes expressed in mature, photosynthetically active leaves, and senescing leaves, respectively, were analyzed. Shown are genes that were successfully overexpressed (*UPPERCASE*) or repressed/knocked out (*lowercase and underlined*) and resulted in increased biomass (B), seed yield (Y), seed protein (SP), and/or plant N use efficiency (NUE) at varying N supply. As indicated by framed white, green, purple, or blue backgrounds, the transgenic strategies either used (1) tissue-specific gene manipulation, (2) concurrent expression of a particular gene in different source or sink tissues, (3) co-expression of two genes in root and shoot tissues (*OsAMT1;2* and *OsGOGAT1*, Lee et al., 2020a), or (4) constitutive gene expression throughout the plant. Plants were grown under high (HN), moderate (MN), sufficient (SN; N supply was not specified), or low N (LN). For native/endogenous gene expression only gene names are provided, while the expression of non-native genes is indicated by the gene name and the common name of the transgenic plant species. Positive results were achieved for genes of a nitrate reductase (*OsNR2*, Gao et al., 2019b), glutamine synthetases (*NtGS1*, Oliveira et al., 2002; *ZmGln1-3*, Martin et al., 2006; *SbGS1*, Urriola and Rathore, 2015; bacterial *GS I*, Zhu et al., 2015; *DvGS2*, Zhu et al., 2014), glutamate synthases (*MsNADH-GOGAT*, Chichkova et al., 2001; *OsGOGAT1*, Lee et al., 2020a), asparagine synthetases (*OsASN1*, Lee et al., 2020b; *E. coli EcasnA*, Seiffert et al., 2004), nitrate (*OsNRT1.1A*, Wang et al., 2018b; (*At*)*NC4N*, a synthetic gene construct based on *Arabidopsis* gene sequences, Chen et al., 2020), and amino acid transporters (*PsAAP1*, Perchlik and Tegeder, 2017) as well as for glutamate dehydrogenases (*Trichurus* sp. *TrGDH*, Du et al., 2019; *C. ehrenbergii CeGDH*, Zhou et al., 2015b; *P. cystidiosus PcGDH*, Zhou et al., 2014; *E. cheralieri EcGDH*, Tang et al., 2018; *E. coli EcgdhA*, Ameziane et al., 2000; Lightfoot et al., 2007; *Aspergillus nidulans AngdhA*, Egami et al., 2012) and autophagy-related proteins (*AtATG8*, Chen et al., 2019; *OsATG8a*, Yu et al., 2019; *OsATG8b*, Zhen et al., 2019a, Fan et al., 2020; *OsATG8c*, Zhen et al., 2019b). Pathways of N (re)assimilation, AA synthesis and conversion (AA_(1)_ to AA_(2)_), organelle and N metabolite degradation, as well as N phloem loading and xylem-phloem exchange (arrows with circle) are shown. Arrows in xylem and phloem indicate possible directions of translocation. For additional information see manuscript text.

Root uptake of inorganic N forms often dominates and requires the function of nitrate and ammonium transporters, respectively. Nitrate uptake is achieved by transporters of the NRT1 and NRT2 family, respectively (Williams and Miller, 2001; Tsay et al., 2007; Krapp et al., 2014; Fan et al., 2017). Studies on natural variation and genetic manipulation of *NRT1* or *NRT2* expression have resolved that NRT function in roots influences N acquisition, plant growth, and seed development (Huang et al., 1999; Cerezo et al., 2001; Remans et al., 2006; Li et al., 2007; Hu et al., 2015; Chen et al., 2016, 2017; Wang et al., 2020). To date, successes in improving NUE under both high and low N have mainly been achieved when *NRT* transporters were constitutively overexpressed (Fang et al., 2013; Fan et al., 2016a,b; Feng et al., 2017; c.f. Wang et al., 2018a) or expressed in both root and leaf tissues (Figures 1, 2; Hu et al., 2015; Chen et al., 2016, 2017; Wang et al., 2018b). While it remains to be determined if root-specific overexpression of *NRT*s may be sufficient for enhancing N uptake, research suggests that downstream nitrate transport processes may additionally or dominantly contribute to the observed increases in NUE. Indeed, recent work in Arabidopsis (*Arabidopsis thaliana*), tobacco (*Nicotiana tabacum*), and rice (*Oryza sativa*) showed that overexpression of the hyperactive chimeric nitrate transporter *AtNC4N* in the phloem of old leaves results in improved NUE under high and low N status (Chen et al., 2020). This study also supports the fact that during leaf senescence, besides amino acids and peptides (Winter et al., 1992; Feller and Fischer, 1994; Masclaux et al., 2000), nitrate recycling from the vacuole and subsequent source-to-sink partitioning contribute to sink N supply. In line with this, increased expression of the vacuolar *OsNRT1.1A/OsNPF6.3* nitrate transporter either throughout rice plants, or in the epidermis and vascular tissues of roots and parenchyma cells of culms and leaf sheaths resulted in increased grain yield and NUE under high and low N supply, due to improved N utilization for grain development (Wang et al., 2018b). As nitrate transport, related N assimilation, and amino acid partitioning processes are highly intertwined (Tegeder and Rentsch, 2010; Tegeder, 2012, 2014; Tegeder and Masclaux-Daubresse, 2018), it would be interesting to examine if in *AtNC4N* and *OsNRT1.1A* overexpressors co-regulated by changes in amino acid pools and transport also add to the observed increases in seed yield, seed N content, and NUE (Wang et al., 2018b; Chen et al., 2020).

Uptake of ammonium is regulated by ammonium transporters (AMTs) as well as aquaporins or cation channels (Glass et al., 2002; Sonoda et al., 2003; Jahn et al., 2004; Loqué et al., 2005; Guo et al., 2007; Bárzana et al., 2014). Although overexpression of *OsAMT1;1* in rice under the control of a ubiquitin promoter resulted in increased seed yield under suboptimal and optimal N conditions (Figure 1; Ranathunge et al., 2014), numerous studies on genetic manipulation of ammonium uptake had rather limited success (Meister et al., 2014; Li et al., 2017; Zhang et al., 2020a). For example, approaches using *CaMV-35S* promoter-*OsAMT1;1* or *OsAMT1;3* constructs in rice resulted in increased ammonium acquisition but had no effect on plant performance (Kumar et al., 2006) or even showed a decrease in biomass and seed yield (Bao et al., 2015). Generally, the use of ammonium transporters as targets for improving N uptake and utilization may be problematic since changes in cellular ammonium pools or excess ammonium can be toxic for the plant cell (Britto and Kronzucker, 2002; Bittsánszky et al., 2015). Nevertheless, detrimental effects of ammonium can potentially be avoided if downstream N assimilation keeps up with its increased uptake. This is supported by a study in rice using activation tagging lines, where *OsAMT1;2* and *OsGOGAT1* genes were simultaneously overexpressed. The modified plants showed an increased grain yield under N-limited growth conditions and improved grain N as well as protein content under both limiting and sufficient N supply (Figures 1, 2; Lee et al., 2020a). Overall, the activation tagging lines were more efficient in N uptake, remobilization, and utilization for sink development and/or N nutrition, clearly presenting an encouraging strategy for improving NUE in rice and most probably other plant species.

## Importance of Amino Acid Uptake and Root-to-Shoot Allocation for NUE

Although the contribution of organic vs. inorganic N acquisition to total root N uptake has not been resolved (Näsholm et al., 2009), it has been demonstrated that plants are highly capable in acquiring organic N, and especially amino acids, via root-localized transporters (Komarova et al., 2008; Paungfoo-Lonhienne et al., 2008; Tegeder and Masclaux-Daubresse, 2018). Such uptake systems are probably of special relevance when amino acid levels are relatively high as observed in soils of organic farming or other cropping systems that rely on manure or compost for N nutrition (Jones et al., 2005a,b; Moran-Zuloaga et al., 2015; Enggrob et al., 2019) or when conservation agriculture is used (Li et al., 2018; Alam et al., 2020). Indeed, it has been suggested that a high proportion of fertilized organic matter compared to mineral N fertilizer results in higher crop N uptake, grain yields, and NUE, with the additional benefit of reduced N losses into the environment (Xia et al., 2017).

Root importers for amino acids include amino acid permeases AAP1 and AAP5, proline transporter ProT2, and lysine-histidine-type transporters LHT1 and LHT6 (Grallath et al., 2005; Hirner et al., 2006; Lee et al., 2007; Svennerstam et al., 2007, 2008, 2011; Lehmann et al., 2011; Perchlik et al., 2014; Ganeteg et al., 2017; Wang et al., 2019; Guo et al., 2020a,b), and their importance for plant growth has been demonstrated (Tegeder and Masclaux-Daubresse, 2018; Guo et al., 2020a). In rice, constitutive overexpression of *OsAAP1* led to increased N uptake and reallocation, positively affecting tiller number and final grain yield (Figure 1; Ji et al., 2020). Nevertheless, it remains to be examined if and how root-specific overexpression of *OsAAP1* (or other amino acid transporters) influences N uptake and NUE, including under varying soil amino acid concentrations.

For root-to-shoot translocation, amino acids taken up from the soil or synthesized in roots are exported from root endodermal cells, the pericycle, or vascular parenchyma into the apoplast (Tegeder, 2014) via usually multiple acids move in and out transporters (UmamiTs; Ladwig et al., 2012; Müller et al., 2015; Besnard et al., 2016) and subsequently loaded into the xylem. Most of the xylem amino acids are then moved to photosynthetically active, transpiring leaves (Miflin and Lea, 1977; Schobert and Komor, 1990) followed by their import into the mesophyll cells, which in Arabidopsis is mediated by LHT1 (Hirner et al., 2006; Liu et al., 2010; Svennerstam et al., 2011). It has not yet been analyzed if and how transporter function in root amino acid export/xylem loading and import into leaf cells affects N uptake and allocation to the shoot, and NUE.

Not all amino acids that are transported out of the root are moved to leaves. Especially during the vegetative phase, up to 21% of the organic N may be retrieved from the transpiration stream along the pathway (van Bel, 1984) for metabolism (Bailey and Leegood, 2016), establishment of N storage pools (Streeter, 1979; Millard, 1988), or xylem-to-phloem transfer to directly supply growing sinks with N (Pate et al., 1975; van Bel, 1984, 1990; Dickson et al., 1985). In Arabidopsis, transporters of the AAP family have been shown to be involved in the removal of amino acids from the xylem (i.e., AtAAP6; Hunt et al., 2010) and their loading into the phloem (i.e., AtAAP2, AtAAP3, and potentially AtAAP5; Okumoto et al., 2004; Zhang et al., 2010; Tegeder and Ward, 2012; Perchlik and Tegeder, 2018). *GmAAP6a* and *OsAAP6* have been overexpressed in soybean and rice, respectively, using the *CaMV-35S* promoter (Figures 1, 2; Peng et al., 2014; Liu et al., 2020). In the transgenic soybean plants, total seed N as well as protein content were improved under sufficient and low N conditions, respectively. Similarly, the rice overexpressors showed improvement in grain protein content under relatively high N supply (Peng et al., 2014). When knocking out *AtAAP2*, which functions in the xylem-phloem exchange of amino acids in Arabidopsis, more N was allocated to leaves resulting in increased photosynthetic N use efficiency [pNUE, i.e., the ratio of CO_2_ fixation to organic N per leaf area (Poorter and Evans, 1998)], carbon fixation, and sucrose transport from source-to-sink independent of the N supply (Perchlik and Tegeder, 2018). The *Ataap2* mutants displayed increased growth, seed yield, and seed carbon storage pools under high, limiting, and highly deficient N supply (Figures 1, 2). NUE efficiency was improved due to increased NUpE, while NUtE was not changed leading to a decrease in N content in individual seeds. In these *Ataap2* plants, increased amounts of N were trapped in the straw/stubble tissue, indicating that remobilization was not adjusted and presented a bottleneck for seed storage protein accumulation (Perchlik and Tegeder, 2018). Similar to the Arabidopsis AAPs, both rice OsAAP3 and OsAAP5 seem to be, at least to some extent, expressed in the vasculature and may function in amino acid exchange between the xylem and phloem (Lu et al., 2018; Wang et al., 2019). Indeed, rice *OsAAP3* and *OsAAP5* RNAi lines also showed significant improvements under tested sufficient N conditions, including increases in tiller number and grain yield (Figures 1, 2).

## Contribution of Source Leaf Nitrogen Metabolism and Amino Acid Transport Processes to NUE

### Nitrogen Assimilation and NUE

Following uptake, nitrate is reduced in the cytosol to nitrite which is transported into the plastid for further reduction to ammonium (Lam et al., 1996; Tegeder and Rentsch, 2010; Liu et al., 2015). The two-step reduction is achieved by nitrate reductase (NR) and nitrite reductase (NiR), respectively. NR catalyzes the main regulatory step in the N assimilation process and its activity is highly regulated by factors such as nitrate, light, or water availability (Beevers and Hageman, 1969; Djennane et al., 2002; Chamizo-Ampudia et al., 2017; Mauceri et al., 2020). In addition, amino acids (e.g., glutamine) present signals regulating uptake and assimilation by affecting both the activity of nitrate transporters (Rawat et al., 1999; Vidmar et al., 2000; Thornton, 2004; Miller et al., 2008) and NR (Dzuibany et al., 1998; Migge et al., 2000a; Fan et al., 2006). Overexpression of *NR* or *NiR* has often resulted in increased N uptake, however improvements in seed yield, seed protein level, or NUE have generally not been achieved, potentially due to negative feedback of N-containing metabolites and related transcriptional or post-transcriptional regulation (Crawford, 1995; Crété et al., 1997; Good et al., 2004; Li et al., 2020). Nevertheless, recent research in *japonica* rice showed that constitutive expression of *indica OsNR2*, which encodes a NADH/NADPH-dependent NR, confers increased tiller number, grain yield, and NUE at high N supply (Figure 2; Gao et al., 2019a). These effects were further enhanced when *indica OsNR2* and *OsNRT1.1B* were concurrently expressed, indicating that *OsNR2* positively regulates *OsNRT1.1B* and thereby regulates nitrate uptake (Gao et al., 2019a).

Ammonium is assimilated through the coordinated activity of glutamine synthetase (GS) and glutamate synthase (GOGAT) into glutamine and glutamate, respectively, which serve as N donors for the biosynthesis of other amino acids and amides (Chichkova et al., 2001; Brauer et al., 2011; Lea and Miflin, 2011). Two isoforms exist for GS, the cytosolic GS1 and plastidic GS2, which have distinct functions in N assimilation and are encoded by multigenic or single gene families (Miflin and Habash, 2002; Chardon et al., 2012). GS2 is involved in primary N assimilation (i.e., *de novo* amino acid synthesis) as well as re-assimilation of ammonium produced during photorespiration (Cren and Hirel, 1999; Ferreira et al., 2019). GS1 isoforms are mainly involved in N re-assimilation and remobilization, with some exceptions such as AtGLN1;2 in Arabidopsis, which is important for N assimilation under high nitrate supply (Lothier et al., 2011), and OsGS1;1 involved in ammonium assimilation in rice shoots and roots (Kusano et al., 2011). Due to their fundamental role in N metabolism and to improve plant growth and seed yield, *GS1* and *GS2* genes have been overexpressed in numerous species, including in tobacco (Migge et al., 2000b; Fuentes et al., 2001; Oliveira et al., 2002; Wang et al., 2013a), Arabidopsis (Zhu et al., 2014; Hu et al., 2015), Sorghum bicolor (sorghum; Urriola and Rathore, 2015), *Zea mays* (maize; Martin et al., 2006), *Triticum aestivum* (wheat; Habash et al., 2001; Hu et al., 2018), *Hordeum vulgare* (barley; Gao et al., 2019b), and rice (Cai et al., 2009; Brauer et al., 2011; Bao et al., 2014). While in some cases no or negative effects on plant performance have been reported (Migge et al., 2000b; Cai et al., 2009; Brauer et al., 2011; Bao et al., 2014; c.f. Thomsen et al., 2014; Garnett et al., 2015; Sweetlove et al., 2017), other studies have shown improvements in seed yield, NUE, NUpE, or NUtE for both GS1 and GS2, and repeatedly at high and low N supply (Figure 2; Oliveira et al., 2002; Martin et al., 2006; Brauer et al., 2011; Zhu et al., 2014; Hu et al., 2015, 2018; Urriola and Rathore, 2015; Gao et al., 2019b). For example, increased expression of *HvGS1-1* under the control of its own promoter in barley led to improved grain yields and NUE under high and low N conditions (Gao et al., 2019b). Zhu et al. (2015) constitutively expressed bacterial *GS I* genes from *Klebsiella* sp. D1-5 and *Lactococcus* sp. Zjy3, respectively, in Arabidopsis and showed, for both genes, improved plant growth and NUE at low and sufficient N. Similarly, overexpression of *TaGS2-2Ab* in wheat using its endogenous promoter resulted in higher seed yield and NUE when N supply was high and low, due to increased N uptake and remobilization (Hu et al., 2018).

Compared to GS, relatively few studies have looked at altering the expression of genes encoding for plastid-localized ferredoxin-dependent (Fd-GOGAT) or NADH-dependent (NADH-GOGAT) GOGAT (Good et al., 2004; Xu et al., 2012). While Fd-GOGAT is primarily involved in the assimilation of ammonium that originates either from nitrate reduction or leaf photorespiration, NADH-GOGAT is highly expressed in non-photosynthesizing cells and has a distinct function in non-photorespiratory ammonium reduction (Somerville and Ogren, 1980; Lancien et al., 2002; Lee et al., 2020). Using Arabidopsis, barley, rice, or *Medicago sativa* (alfalfa) mutants, essential roles in plant growth and seed development have been demonstrated for both Fd-GOGAT and NADH-GOGAT (Somerville and Ogren, 1980; Kendall et al., 1986; Schoenbeck et al., 2000; Lancien et al., 2002; Potel et al., 2009; Tamura et al., 2010; Yang et al., 2016; Zeng et al., 2017). However, relatively few studies manipulated *GOGAT* expression to enhance seed yield and NUE, and those focused on *NADH-GOGAT* with rather moderate outcomes. For instance, *OsNADH-GOGAT* overexpression in rice using its endogenous promoter only resulted in increased grain weight at low N fertilization (Yamaya et al., 2002). Further, constitutive expression of *MsNADH-GOGAT* showed higher biomass production in tobacco when N was sufficient (Figure 2; Chichkova et al., 2001), whereas in maize, overexpression of *ZmNADH-GOGAT* resulted in a lower shoot biomass and no changes in kernel yield (Cañas et al., 2020). Interestingly, and as pointed out above, combined overexpression of *OsAMT1;2* and *OsNADH-GOGAT1* in rice improved NUE at both sufficient and low N conditions (Figure 2). At sufficient N, the transgenic lines achieved higher seed protein levels without altering seed yield, while at low N both seed protein and yield were improved (Lee et al., 2020a). The results suggest that by increasing both N uptake and N assimilation, negative intrinsic effects that might happen when only *AMT* (see above) or *GOGAT* is overexpressed (e.g., through unbalanced pools of N metabolites) may be avoided (Lee et al., 2020a).

### Amino Acid Synthesis and NUE

Relatively few studies have described improvements in NUE by altering the expression of genes related to amino acid synthesis, with the exception of asparagine synthetase (*ASN*) and alanine aminotransferase (*AlaAT*) (Lea and Azevedo, 2007; McAllister et al., 2012; Xu et al., 2012). Both asparagine and alanine are major long-distance transport forms of N in certain plants (Atkins et al., 1975; Winter et al., 1992; Miflin and Habash, 2002; Lea et al., 2007). Asparagine is synthesized through ASN, which functions in both N assimilation and remobilization (Sieciechowicz et al., 1988; Lam et al., 1995, 1996; Gaufichon et al., 2013, 2017; Moison et al., 2018; Lee et al., 2020b). Constitutive overexpression of *OsASN1* was somewhat successful in rice and showed increased seed N content and NUE under limiting and sufficient N treatments, as well as additional improvements in shoot biomass and seed yield at low N (Figure 2; Lee et al., 2020b). On the other hand, expression of *Escherichia coli EcasnA* in *Brassica napus* (canola) seems to only be successful at high N status (Seiffert et al., 2004). AlaAT is involved in alanine synthesis and catabolism, and the constitutive or root-specific expression of barley *HvAlaAT* resulted in improved seed yields in wheat (Peña et al., 2017), or in canola (Good et al., 2007) and rice (Shrawat et al., 2008) (Figure 1). In addition, NUE was increased in rice, canola, and Saccharum officinarum (sugarcane) when *HvAlaAT* expression was targeted to roots and engineered plants were exposed to high N (Beatty *et al*., 2013) and/or low N (Good et al., 2007; Snyman et al., 2015). How AlaAT function in roots affects seed yield and NUE is unclear, as it seems to not be vital for plant N metabolism and source-to-sink partitioning (Lam et al., 1996; Miyashita et al., 2007; Beatty et al., 2013; McAllister and Good, 2015).

### Remobilization of Source Nitrogen and NUE

A relatively high portion of N that is delivered to seeds or other storage sinks may derive from the recycling or remobilization of N in source leaves, rather than directly from root uptake (Cliquet et al., 1990; Patrick and Offler, 2001; Masclaux-Daubresse et al., 2010; Masclaux-Daubresse and Chardon, 2011). This includes nitrates, amino acids, and ureides that are temporarily stored in vacuoles during the vegetative stage (Staswick, 1994; Diaz et al., 2008; Masclaux-Daubresse and Chardon, 2011), as well as N from proteins and organelles, especially from chloroplast degradation (Havé et al., 2017). Overall, the degree of N remobilization in source tissues and its allocation to sinks is assumed to have a significant impact on grain filling, yield, and NUE, while decreasing the residual N in vegetative tissues at harvest (Masclaux-Daubresse and Chardon, 2011). N remobilization and reassimilation occurs especially during leaf senescence and mainly involves the activity of the cytosolic GS1, which has been shown to affect NUE (see above; Masclaux-Daubresse et al., 2010; Avila-Ospina et al., 2014; Havé et al., 2017). Glutamate dehydrogenase (GDH) may also have some role in N assimilation (Lam et al., 1996; Melo-Oliveira et al., 1996; Good et al., 2004; Lea and Miflin, 2011), but it seems to predominantly function in glutamate deamination and ammonium supply for GS1 (Havé et al., 2017; Moison et al., 2018) during N remobilization or when C is limited (Robinson et al., 1991; Masclaux-Daubresse et al., 2006; Fontaine et al., 2012; Grzechowiak et al., 2020). Efforts to enhance plant performance and NUE by overexpressing *GDH* have been inconclusive. Studies have been unsuccessful when using constitutive overexpression of tomato *Slgdh-NAD;B1* in tobacco (Purnell et al., 2005), *Nicotiana plumbaginifolia GDHA* and *GDHB* in *N. tabacum* (Tercé-Laforgue et al., 2013), and *GDH* from fungi *Sclerotinia sclerotiorum* and *Magnaporthe grisea* in rice (Du et al., 2014; Zhou et al., 2015a). On the other hand, there is good evidence that constitutive overexpression of *GDH* can also lead to increased growth, seed yield, and/or NUE at different N regimes (Figure 2). Specifically, expression of *EcgdhA* from *E. coli* in tobacco and maize resulted in increased biomass under sufficient N conditions (Ameziane et al., 2000) and increased seed yield under high N supply (Lightfoot et al., 2007), respectively. Further, expression of *AngdhA* from *Aspergillus nidulans* in potato (*Solanum tuberosum*) caused increased photosynthesis rates, tuber numbers and tuber dry weight, as well as improved NUE at moderate and low N levels (Egami et al., 2012). In rice, increases in NUE and/or grain yield were achieved at low N when fungal *GDH* from *Cylindrocarpon ehrenbergii* (*CeGDH*) or *Eurotium cheralieri* (*EcGDH*) were expressed (Zhou et al., 2015b; Tang et al., 2018). In addition, improvements in rice grain yield and weight, or in grain storage protein levels were obtained at low and high N as well as without N fertilization by using fungal *GDH*s from *Trichurus* sp. (*TrGDH*, Du et al., 2019) and *Pleurotus cystidiosus* (*PcGDH*, Zhou et al., 2014), respectively. Why constitutive overexpression of *GDH* was successful in some but not all studies is unclear, but it could potentially result from the genes and plant species that were analyzed. Also, a leaf-specific engineering approach may help to more reliably promote source N remobilization through GDH and enhance source-to-sink N allocation.

Autophagy, an essential mechanism for the degradation of proteins, organelles, and cytosolic macromolecules, is enhanced during leaf senescence and strongly contributes to N remobilization and source-to-sink transport of N (Suzuki and Ohsumi, 2007; Avila-Ospina et al., 2014; Chen et al., 2019). Altering expression of autophagy-related proteins (ATGs) has been a successful strategy to enhance N remobilization in Arabidopsis and rice (Figure 2; Chen et al., 2019; Yu et al., 2019; Zhen et al., 2019a,b; Fan et al., 2020). Depending on the study, the outcome was increased plant growth, seed yield, or seed N and protein levels (Figure 2). In Arabidopsis, constitutive overexpression of *AtATG8* resulted in higher seed protein levels when transgenic plants were grown with moderate N supply (Chen et al., 2019), while *OsATG8b* expression led to increased biomass, seed yield, and NUE at moderate and low N (Zhen et al., 2019a). In rice, shoot biomass, seed yield, and protein levels, as well as NUE were improved when *OsATG8a* was overexpressed and plants were exposed to high N nutrition, while at moderate N, positive effects were only observed for biomass and seed protein (Yu et al., 2019). Similarly, constitutive expression of *OsATG8b* in rice resulted in more biomass as well as higher grain yield and protein under sufficient N (Fan et al., 2020), and *OsATG8c* overexpression triggered improvements in shoot biomass, grain yield, and NUE under moderate and low N fertilization (Zhen et al., 2019b). To date, studies on autophagy-related proteins have focused on Arabidopsis and rice, and work is now needed to address their importance for N remobilization and subsequently yield and NUE improvements in other crop species.

### Source Amino Acid Transport Processes and NUE

*De novo* synthesis of most amino acids occurs in plastids (Rolland et al., 2012). Once produced, amino acids are used for plastid function or they are exported from the plastids in support of metabolism in diverse cellular compartments, for transient storage in the vacuole, or for translocation in the phloem to developing sinks (Tegeder and Rentsch, 2010; Taniguchi and Miyake, 2012; Tan et al., 2019). Very little is known about the transport proteins that move amino acids across the chloroplast envelope, as well as between subcellular compartments (Tegeder and Rentsch, 2010; Widhalm et al., 2015; Dinkeloo et al., 2018; Tegeder and Masclaux-Daubresse, 2018; Bouchnak et al., 2019). So far, only two plastidic amino acid transporters have been identified in plants, *Arabidopsis* malate/glutamate translocators (AtDiT2, Renné et al., 2003; Eisenhut et al., 2015) and *Petunia hybrida* cationic amino-acid transporter (PhpCAT, Widhalm et al., 2015). In addition, a few amino acid transporters have been localized to the tonoplast, specifically Arabidopsis AtCAT2, AtCAT4, and vacuolar amino acid transporter AtAVT3 as well as tomato (Solanum lycopersicum) SlCAT9 (Yang et al., 2014; Snowden et al., 2015; Fujiki et al., 2017; Dinkeloo et al., 2018). Further, AtBAC1 and AtBAC2 have been shown to transport basic amino acids across the mitochondrial membrane (Hoyos et al., 2003; Palmieri et al., 2006; Toka et al., 2010). Although the function of these intracellular transporters in NUE has not yet been addressed, their regulatory role is most probably fundamental to the effective use of N, for example through, (i) regulating N levels in the chloroplasts and thereby chloroplast function and photosynthetic N use efficiency, (ii) balancing pools of specific amino acids that may otherwise negatively affect plant metabolism and performance through end-product inhibition or substrate limitations of the respective amino acid synthesis pathways, (iii) making amino acids available for phloem loading and source-to-sink allocation throughout the plant's life cycle, and (iv) regulating transient storage pools of amino acids and their remobilization for reproductive growth.

**Figure 3:**
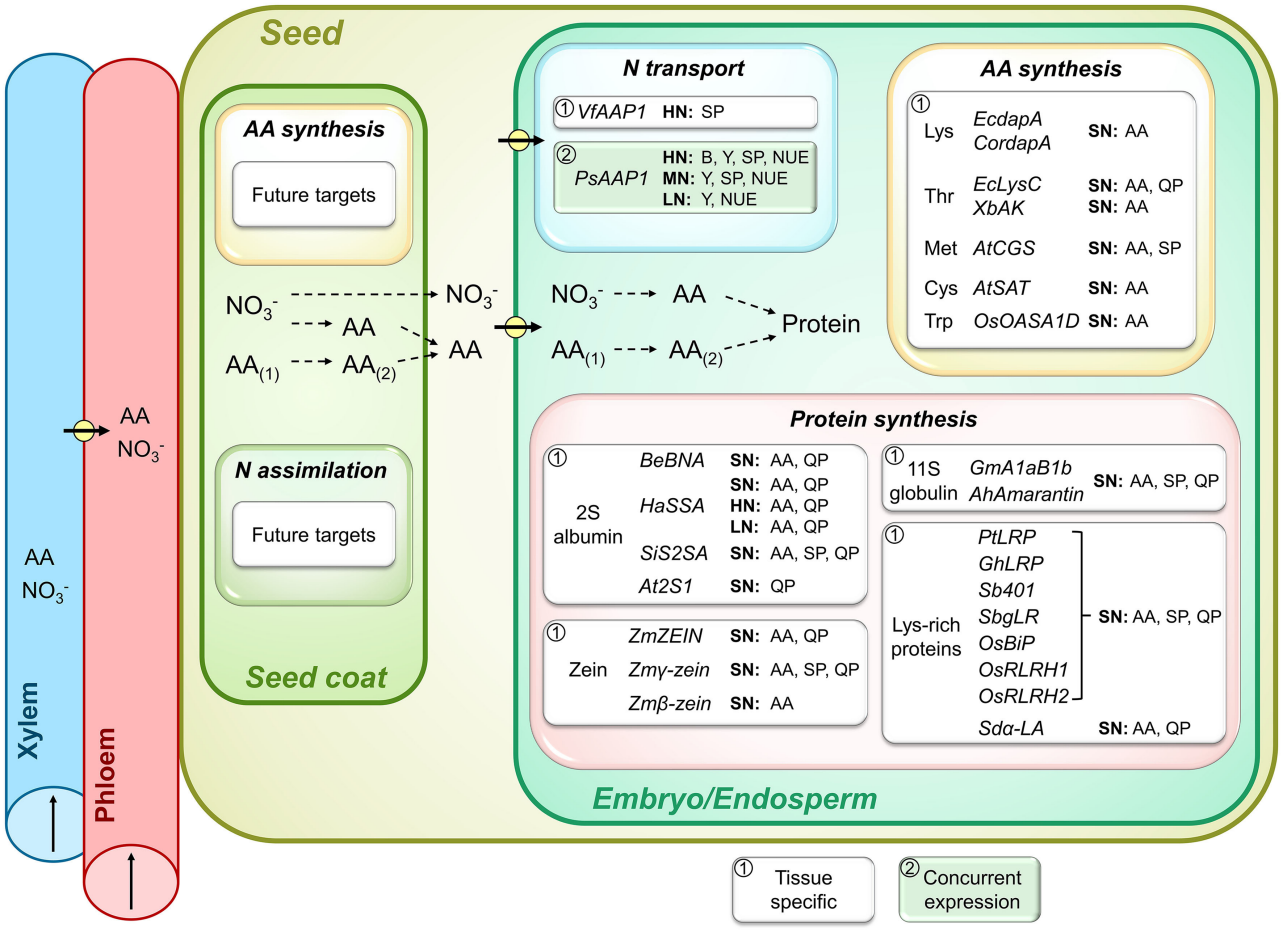
Importance of seed N transport and metabolic processes for seed N accumulation, seed yield, and N use efficiency (NUE). Shown are genes that were successfully overexpressed and resulted in increases in seed levels of specific amino acids (AA), total soluble protein (SP), and specific high-quality proteins (QP), and positively affected shoot biomass (B), seed yield (Y), or NUE. As indicated by a framed white or green background, the transgenic strategies either used (1) seed-specific manipulation or (2) concurrent expression of a particular gene in different source or sink tissues. Plants were grown under high (HN), moderate (MN), sufficient (SN; N supply was not specified), and low N (LN). Pathways of N (re)assimilation, AA synthesis, and conversion (AA_(1)_ to AA_(2)_) in seed coat and embryo as well as N import into the embryo (arrows with circle) and subsequent protein synthesis are shown. Arrows in xylem and phloem indicate the direction of translocation. Positive results were achieved for embryo expression of genes encoding for AA transporters (*VfAAP1*, Rolletschek et al., 2005; Weigelt et al., 2008; *PsAAP1*, Perchlik and Tegeder, 2017), and genes related to synthesis of sulfur-rich (*AtCGS*, Hanafy et al., 2013; Song et al., 2013; Cohen et al., 2014, 2016; *AtSAT*, Tabe et al., 2010) and other essential AA (*EcdapA*, Karachi et al., 1994; Zhu and Galili, 2004; Angelovici et al., 2009; *CordapA*, Falco et al., 1995; Huang et al., 2005; Frizzi et al., 2007; *EcLysC*, Karachi et al., 1993, 1994; *XbAK*, Qi et al., 2011; *OsOASA1D*, Kita et al., 2010) as well as seed storage protein synthesis (*BeBNA*, Altenbach and Simpson, 1989; Altenbach et al., 1992; Pickardt et al., 1995; Saalbach et al., 1995; Demidov et al., 2003; *HaSSA*, Molvig et al., 1997; Tabe and Droux, 2002; Hagan et al., 2003; Chiaiese et al., 2004; *SiS2SA*, Lee et al., 2003, 2005; *At2S1*, De Clercq et al., 1990; *ZmZEIN*, Dinkins et al., 2001; *Zm*γ*-zein*, Li et al., 2005; *Zm*β*-zein*. Guo et al., 2020c; *GmA1aB1b*, Takaiwa et al., 1995; Katsube et al., 1999; Momma et al., 1999; *AhAmarantin*, Rascón-Cruz et al., 2004; *PtLRP*, Liu et al., 2016; *GhLRP*, Yue et al., 2014; *Sb401*, Yu et al., 2004; *SbgLR*, Wang et al., 2013b; *OsBiP*, Yasuda et al., 2009; Kawakatsu et al., 2010; *OsRLRH1* and *OsRLRH2*, Wong et al., 2015; *Sd*α*-LA*, Bicar et al., 2008). Names of genes that were overexpressed are provided, however in contrast to Figures 1, 2, the transgenic plant species are not indicated as the respective genes have often been overexpressed in multiple species. See the manuscript text or listed references for more detailed information.

Phloem loading of amino acids represents a bottleneck in N allocation from source leaves to sink (Tegeder, 2012, 2014) and influences not only sink development but also source physiology including carbon fixation, assimilation, and partitioning to sinks (Tan et al., 2010; Zhang et al., 2015b; Santiago and Tegeder, 2016, 2017). For example, increased expression of the endogenous *amino acid permease 1* (*PsAAP1*) in the pea (*Pisum sativum*) phloem (as well as in the seed cotyledons) resulted in increased amino acid allocation to sinks and co-regulated carbon capture and source-sink allocation. The overall outcome was increased vegetative growth, seed number, and seed protein levels at high N supply (Zhang et al., 2015b). Improvements in seed yield were also achieved when the transgenic plants were exposed to limiting and deficient N levels (Figures 2, 3; Perchlik and Tegeder, 2017). However, factors contributing to the increased NUE varied depending on the N fertilization. Transgenic pea plants exposed to high N soils showed enhanced NUpE, while at low N status NUtE was improved, and at limiting N, both NUpE and NUtE were enhanced. Together, these results support that manipulation of transporters involved in the source-to-sink partitioning of amino acids, and possibly other N metabolites, present a valid strategy to improve NUE. This is further supported by work expressing an *S*-methyl-methionine transporter (Tan et al., 2010) or a transporter for ureides, an important long-distance N transport form in soybean (*Glycine max*), in the phloem (Thu et al., 2020). In both cases, plant growth and seed productivity were improved, at least under high N fertilization.

## Seed Amino Acid Transport Processes and Metabolism as Potential Targets for Improving Seed Quality and NUE

Numerous studies support the fact that the development of vegetative and reproductive sinks is source-limited and depends on the amount of N (and carbon) allocated to sinks (see above; Koch et al., 2003; Chiaiese et al., 2004; Pélissier and Tegeder, 2007; Schmidt et al., 2007; Tan et al., 2010; Zhang et al., 2010, 2015b; Santiago and Tegeder, 2016, 2017; Lu et al., 2020; Thu et al., 2020). On the other hand, seed filling and quality seem to be influenced by seed-localized transport and metabolic processes (Borisjuk et al., 2002; Rosche et al., 2002; Rolletschek et al., 2005; Weigelt et al., 2008; Sanders et al., 2009; Hunt et al., 2010; Ladwig et al., 2012; Pandurangan et al., 2012; c.f. Zhang et al., 2007; Tegeder and Rentsch, 2010; Galili et al., 2016), which may especially be critical when N is limited and individual seeds need to compete for the phloem-derived N.

### Seed Amino Acid Transport Processes

Seed N transporters are thought to regulate N flux toward, and uptake into, the embryo by controlling the rate of allocation, amount, and composition of amino acids (Tegeder, 2012; Dinkeloo et al., 2018; Tegeder and Hammes, 2018). Amino acid transporters are especially needed at the seed coat–embryo interface where symplasmic connections are lacking (Lanfermeijer et al., 1989; Zhang et al., 2007; Ladwig et al., 2012; Tegeder et al., 2013; Besnard et al., 2018; Tegeder and Hammes, 2018). Following efflux from the seed coat into the apoplasm, the amino acids may either be taken up into the embryo/cotyledons via epidermal cells located opposite to the seed coat or they are imported into the storage parenchyma cells from the seed apoplasm (Tegeder et al., 2000, 2007; Offler et al., 2003; Zhang et al., 2007; Tegeder, 2014). In pea and *Vicia narbonensis*, N uptake into the embryo could be increased when overexpressing an amino acid transporter (*VfAAP1*) in the storage parenchyma cells of the cotyledons (Figure 3; Rolletschek et al., 2005; Weigelt et al., 2008). This led to changes in seed metabolism and increased storage protein accumulation under standard N nutrition, including in the field. Further analysis of the transgenic *V. narbonensis* plants revealed that they take up more N for allocation to seeds and over an extended time period, and that carbon fixation and allocation to seeds are improved as well (Götz et al., 2007). Other studies showed that overexpression of the endogenous *PsAAP1* in the epidermal transfer cells of pea cotyledons also resulted in more seed protein when plants were grown at high N fertilization and positively affects carbon metabolism (Zhang et al., 2015b). In those plants, *PsAAP1* was concurrently overexpressed in the phloem, triggering enhanced amino acid partitioning to sinks and increased seed number and yield under high N. When exposed to a 50% reduction in N fertilization, the transgenic *PsAAP1* pea plants still outperformed wild-type control plants with respect to seed yield and NUE, and seed protein levels were unchanged compared to wild-type (Figure 3; Perchlik and Tegeder, 2017). Together, this supports the fact that the higher amounts of phloem amino acids in the transgenic pea plants accommodate both the increased number of seeds as well as improved seed loading of amino acids. It also demonstrates that even under strongly reduced N supply, the simultaneous upregulation of phloem and embryo loading is a successful strategy for avoiding a dilemma generally observed in crop plants (e.g., in soybean, rice, and maize), which is an increase in seed yield at the cost of seed protein levels or vice versa (Simmonds, 1995; Wilcox and Guodong, 1997; Triboi et al., 2006; Rotundo et al., 2009; Gambín and Borrás, 2010). Even under severe N deficiency (80% less N), seed yield was still higher in the *PsAAP1* lines compared to the non-transgenic plants, but at the cost of protein amounts in individual pea seeds (Perchlik and Tegeder, 2017). Nevertheless, total protein yields per plant were always higher in the *PsAAP1* overexpressors, independent of how much N was supplied. It is important to point out that the experiments with *PsAAP1* overexpressors were done with non-nodulated pea plants that solely relied on soil N. It still needs to be determined, if nodulated *PsAAP1* plants, that additionally fix atmospheric N, also outperform wild-type. Together, the above findings support the fact that seed protein levels can be controlled through amino acid transporter functions in the embryo and that their increased activity enables the individual seeds to compete for the phloem N, including under limited N conditions (Figure 3; Rolletschek et al., 2005; Götz et al., 2007; Weigelt et al., 2008; Zhang et al., 2015b; Perchlik and Tegeder, 2017).

### Amino Acid Conversion and Synthesis in Seed Coat and Embryo

While generally a broad spectrum of amino acids is found in the phloem and delivered to seeds, asparagine, glutamine, aspartate, glutamate, alanine, and/or homoserine often dominate, dependent on the plant species, intrinsic factors, and environmental cues (Urquhart and Joy, 1982; Fisher and Macnicol, 1986; Winter et al., 1992; Lohaus et al., 1995; Gattolin et al., 2008). To provide a balanced pool of amino acids and accommodate seed function, amino acid catabolism, N re-assimilation, and amino acid synthesis takes place, either in the seed coat or developing embryo (Figure 3; Rainbird et al., 1984; Ranocha et al., 2001; Lee et al., 2008; Galili et al., 2016; Amir et al., 2018; Tegeder and Masclaux-Daubresse, 2018; Wang et al., 2018c). Amino acids that seem to be primarily produced in the seed coat and released into the seed apoplast are alanine, glutamine, threonine, serine, and valine (Fisher and Macnicol, 1986; Murray, 1987; Rochat and Boutin, 1991; Gallardo et al., 2007). In addition, the sulfur-containing, non-protein amino acid S-methylmethionine is converted to methionine in the seed coat (Ranocha et al., 2001; Gallardo et al., 2007; Lee et al., 2008). Why amino acid metabolism occurs in the seed coat is uncertain, but the conversion of amino acids in the maternal tissue may be important for creating steep concentration gradients for specific amino acids to facilitate their fast movement toward the embryo or to accommodate amino acid transporters involved in embryo uptake of the respective substrate(s). We are not aware of studies examining N metabolism in the seed coat with consequences for seed yield, NUE, or seed protein accumulation, with the exception of work done by Pandurangan et al. (2012). They observed that during soybean seed development asparagine synthetase (ASN) levels in the seed coat decrease while those of asparaginase (ASPGB), involved in asparagine catabolism, increase. Further, ASPGB1a levels and activity were higher in seed coats of a soybean cultivar associated with high seed protein concentrations, suggesting enhanced N flux toward protein synthesis compared to a low-protein cultivar. Overall, the work suggests that seed coat asparagine metabolism may control N partitioning to developing embryos and storage protein accumulation, and could be an important trait for the genetic improvement of seed proteins, at least in soybean.

Following amino acid uptake into the embryo, conversion and transamination reactions also take place in the filial tissue to produce the amounts and spectrum of amino acids required for embryo growth and metabolism, and to produce seed storage proteins (Weber et al., 2005; Tegeder and Rentsch, 2010; Amir et al., 2018). Essential amino acids including aliphatic, aromatic, basic and hydroxyl-group, and sulfur-containing amino acids are critical for human nutrition, but tend to be present in low amounts in many crop species. To achieve higher seed nutritional quality, many studies have therefore targeted genes involved in synthesis of the essential amino acids, often by using seed-specific promoters (Figure 3; Karachi et al., 1993, 1994; Lee et al., 2001; Zhu and Galili, 2004; Kita et al., 2010; Qi et al., 2011; Song et al., 2013; Cohen et al., 2014, 2016; c.f. Galili and Amir, 2013; Wang et al., 2017; Amir et al., 2018). For example, recent work showed that overexpression of cystathionine γ-synthase genes (*AtCGS*) involved in regulating carbon flow toward methionine synthesis (Amir et al., 2002) resulted in improved levels of free methionine in legume and Arabidopsis seeds (Hanafy et al., 2013; Song et al., 2013; Cohen et al., 2014) and an increased usage of methionine and other amino acids for protein synthesis in tobacco and Arabidopsis seeds (Matityahu et al., 2013; Cohen et al., 2014, 2016). Further, free cysteine levels could be improved in seeds of narrow leaf lupin (*Lupinus angustifolius*) by manipulating a serine acetyltransferase (*AtSAT*) (Tabe et al., 2010), and lysine content was increased by expressing feedback insensitive dihydrodipicolinate synthases (*DHDPS*) in Arabidopsis (*E. coli EcdapA*, Zhu and Galili, 2004; Angelovici et al., 2009), tobacco (*EcdapA*, Karachi et al., 1994), canola and soybean (*Corynebacterium glutamicum CordapA*, Falco et al., 1995), rice (*Zmdhps*, Lee et al., 2001), and maize seeds (*CordapA*, Huang et al., 2005; Frizzi et al., 2007). Other studies overexpressed aspartate kinases in soybean (*EcAK*, Karachi et al., 1993, 1994; *Xenorhabdus bovienii XbAK*, Qi et al., 2011) or an anthranilate synthase subunit in rice (*OsOASA1D*, Kita et al., 2010) and successfully enhanced free threonine and tryptophan levels, respectively. Most approaches for altering amino acid synthesis in seeds not only resulted in an increase of the targeted amino acid, but also positively affected levels of other free amino acids (Karachi et al., 1994; Zhu and Galili, 2004; Huang et al., 2005; Qi et al., 2011; Hanafy et al., 2013; Song et al., 2013; Cohen et al., 2014, 2016; Galili et al., 2016; Wang et al., 2017, 2018c). However, unfortunately only a few studies analyzed total soluble protein levels and/or reported increases in seed proteins (Matityahu et al., 2013; Song et al., 2013; Cohen et al., 2014, 2016) or found changes in the amounts of high-quality proteins, such as the methionine-rich globulins and albumins (Figure 3; Karachi et al., 1993, 1994; Angelovici et al., 2009; Cohen et al., 2014, 2016). Seemingly, information on if or how changes in seed amino acid synthesis can affect seed yield and NUE is also lacking.

### Protein Synthesis in the Embryo

Seeds of many crop species contain relatively few amounts of high-quality proteins, such as the sulfur-rich albumins and legumin-like globulins. Much research has been done to enhance seed N use and increase the amount and quality of proteins by seed-specific overexpression of storage protein genes (Figure 3; c.f. Ufaz and Galili, 2008; Galili and Amir, 2013; Häkkinen et al., 2018). For example, 2S albumin genes (*BNA, SA*, or *2SA*) have been successfully expressed in seeds of rice (*Sesamum indicum SiS2SA*, Lee et al., 2003, 2005; *Helianthus annuus HaSSA*, Hagan et al., 2003), tobacco (*Bertholletia excelsa BeBNA*, Altenbach and Simpson, 1989; Saalbach et al., 1995; *At2S1*, De Clercq et al., 1990), canola (*At2S1*, De Clercq et al., 1990; *BeBNA*, Altenbach et al., 1992), *Vicia narbonensis* (*BeBNA*, Pickardt et al., 1995; Saalbach et al., 1995; Demidov et al., 2003), *Cicer arietinum* (chickpea; *HaSSA*, Chiaiese et al., 2004), narrow leaf lupin (*HaSSA*, Molvig et al., 1997; Tabe and Droux, 2002), and Arabidopsis (*At2S1*, De Clercq et al., 1990). Further methionine-rich zein proteins from maize could be produced in soybean seeds (*ZmZEIN*, Dinkins et al., 2001; *Zm*γ*-zein*, Li et al., 2005; *Zm*β*-zein*, Guo et al., 2020c), and 11S globulins from soybean or amaranth (*Amaranthus hypochondriacus*) were expressed in rice (*GmA1aB1b*, Katsube et al., 1999; Momma et al., 1999) and tobacco (*GmA1aB1b*, Takaiwa et al., 1995), and maize (*AhAmarantin*, Rascón-Cruz et al., 2004), respectively. Besides the successes in increasing the methionine/sulfur-rich proteins, only a few studies reported additional positive effects on total seed proteins (Figure 3; Momma et al., 1999; Lee et al., 2003; Rascón-Cruz et al., 2004; Li et al., 2005). If and how varying N fertilization influences seed quality and protein levels, or seed protein yields of transgenic plants expressing storage protein genes has generally not been addressed, with the exception of work by Chiaiese et al. (2004). The authors showed that chickpea plants expressing a sunflower 2S albumin gene (*HaSSA*) achieved higher seed albumin levels under high and low N supply, while seed yield was not changed at high N but decreased at low N fertilization. However, since sulfur was sequestered in the sulfur-rich protein, the negative effects were mainly attributed to sulfur rather than N deficiency (Chiaiese et al., 2004).

Seed quality is also influenced by the lysine content of proteins, and so-called lysine-rich proteins (LRP) have been successfully increased in rice (Liu et al., 2016) and maize (*Solanum berthaultii Sb401*, Yu et al., 2004; *Psophocarpus tetragonolobus PtLRP*, Liu et al., 2016; *SbgLR*, Wang et al., 2013b; *Gossypium hirsutum GhLRP*, Yue et al., 2014; Figure 3). Other proteins with high lysine content have also been used to improve the protein quality of seeds, including a binding protein (*OsBiP*, Yasuda et al., 2009; Kawakatsu et al., 2010) and lysine-rich histone proteins (*OsRLRH1* and *OsRLRH2*, Wong et al., 2015) in rice, and a porcine α-lactalbumin gene in maize (*Sus domesticus Sd*α*-LA*, Bicar et al., 2008). In addition, the high-lysine lines displayed increased total seed protein levels, while seed yield was not analyzed (Figure 3). The increases in both specific and total seed proteins support enhanced seed N supply, probably due to increased sink strength (MacKown et al., 1992; Rolletschek et al., 2005; Götz et al., 2007) and elevated N assimilate availability for protein synthesis, which is reinforced by higher seed amino acid levels observed in numerous studies (Figure 3; Momma et al., 1999; Rascón-Cruz et al., 2004; Yasuda et al., 2009; Kawakatsu et al., 2010; Liu et al., 2016).

Improvements in quality proteins have generally been observed at sufficient or high N fertilization, and it is not clear if the transgenic plants maintain their advantage at low N supply, and if and how total seed protein and yield would be affected under non-optimal N conditions. Since seed protein concentrations generally depend on N assimilate availability (Rolletschek et al., 2005; Weigelt et al., 2008; Rotundo et al., 2009; Pandurangan et al., 2012; Santiago and Tegeder, 2016), it also remains to be evaluated if increasing amino acid source-to-sink allocation (c.f. Zhang et al., 2015b; Perchlik and Tegeder, 2017), in addition to upregulation of seed amino acid (see above) or protein synthesis, can further improve seed protein yields and quality, even under low N status, and leads to improved NUE.

## Conclusion and Future Perspectives

The movement of N from the soil to final sink cells involves numerous transport and metabolic processes in source and sink, including (i) N uptake and partitioning within cells and over short and long distances, and (ii) N (re)assimilation, amino acid synthesis and catabolism, and protein synthesis. Since any of these processes could potentially present a bottleneck for the efficient use of N for biomass and seed production, associated key genes have been targets for improving plant performance and NUE, but, as reviewed above, with varying success (Figures 1–3). The inconsistent outcomes might, in part, be caused by unwanted pleiotropic effects due to the use of constitutive promoters, an imbalance in N metabolite pools triggering negative feedback inhibition of N uptake or metabolism, substrate limitations for amino acid synthesis, or post-transcriptional and post-translational regulation (Thomsen et al., 2014; Takabayashi et al., 2016; Osanai et al., 2017; Sweetlove et al., 2017; Gao et al., 2019b; Fernie et al., 2020). To circumvent such detrimental effects and to improve NUE, future research may consider the selection or design of “super promoters” for genetic manipulation that allow tissue or cell-specific, developmentally regulated and enhanced gene expression. Such promoters could, for example, be used for the overexpression of amino acid transporters or synthesis genes to drive N uptake from the soil throughout the vegetative and reproductive phases, or to accelerate source N remobilization and leaf export during leaf senescence. Other research studies could consider a “gene pyramiding approach” (Halpin, 2005; Li et al., 2010; Naqvi et al., 2010; McAllister et al., 2012; Lee et al., 2020a). While selective metabolism and transporter genes have been successful targets to improve NUE in some plant species, at least under high N nutrition (see above), simultaneous manipulation of several genes may be required for a broader approach to improve NUE at high and low N status. For example, improvements in N assimilation or amino acid synthesis may only be successful if metabolic and transport processes are coordinated through concurrent manipulation of two or more genes (c.f., Lee et al., 2020a). Further, one gene could be simultaneously manipulated in root and shoot tissues, or source and sink (c.f., Zhang et al., 2015a; Lee et al., 2020a). For instance, to improve N flux from source to sink and seed N supply, leaf N export and phloem loading, as well as seed transport processes including embryo loading, will need to happen at a high rate and throughout sink development (c.f., Perchlik and Tegeder, 2017; Tegeder and Hammes, 2018). Approaches to improve crop productivity and NUE should also take into account regulatory aspects of N assimilation and transport, as well as the sensing and signaling mechanisms involved (Wang et al., 2018a; Chen et al., 2020; Feng et al., 2020; Han et al., 2020; Vidal et al., 2020). Consideration should further be given to the tight interaction between carbon, N, and sulfur metabolism and their transport processes (Zhang et al., 2015b; Tegeder and Masclaux-Daubresse, 2018; Kopriva et al., 2019; Fernie et al., 2020).

NUE is a complex polygenic trait making its genetic dissection and engineering challenging. In fact, despite the notable progress that has been made in understanding plant NUE, successful agronomic applications of engineered crop plants with altered N transport and metabolism or other physiological functions have not yet been achieved. To better grasp the genetic basis of NUE for genetic manipulation, future strategies might take greater advantage of the available natural variation for NUE between and within crop species (Hawkesford and Griffiths, 2019; Swarbreck et al., 2019). For example, using genetic association studies or genomic selection, in conjunction with high-throughput phenotyping, large crop populations, or different cultivars could be screened to discover further genes associated with improved NUE (Bhat et al., 2016; van Bueren and Struik, 2017; Nguyen and Kant, 2018; York, 2019; Sinha et al., 2020). Alternatively, or in addition, comparative transcriptome and proteome studies will allow for identification of fundamental NUE genes (Simons et al., 2014; Li et al., 2020; Zhang et al., 2020b). Importantly, to develop improved crop cultivars for a range of growing regions, such analyses will have to be done with plants exposed to a variety of locations, latitudes, soil conditions, and varying N regimes and agricultural systems. These should include systems that do not solely rely on mineral N fertilizer but also consider organic matter for plant N nutrition, like organic farming or conservation agriculture, and the need of amino acid transporters for N uptake. Further, to improve NUE for a sustainable agriculture, future climate and environmental changes will need to be addressed (Nguyen et al., 2017; Kanter et al., 2020). For example, exposure of crop plants to drought stress will not only require adjustments with respect to N uptake, partitioning, and metabolic processes, but also other physiological as well as morphological changes. As it would be extremely challenging to conduct field experiments for conventional breeding, marker-assisted modern breeding, genomic selection, and genetic engineering under numerous scenarios, the use of crop modeling that integrates genetics, environmental factors, and management practices will be a critical tool for making progress in NUE (Zhang et al., 2015a; Messina et al., 2020; Peng et al., 2020). Indeed, interdisciplinary strategies and multiscale collaborations are essential to achieve a more robust and holistic understanding of NUE that can lead to global successes for crop production and a more sustainable agroecological system (c.f., Cassman and Grassini, 2020).

## Author Contributions

All authors listed have made a substantial, direct, and intellectual contribution to the work, and approved it for publication.

## Conflict of Interest

The authors declare that the research was conducted in the absence of any commercial or financial relationships that could be construed as a potential conflict of interest.
